# Vaginal Microbiome Is Associated with Breed and Pregnancy Status in Beef Cattle

**DOI:** 10.3390/ani16060874

**Published:** 2026-03-11

**Authors:** Breno Fragomeni, Sarah M. Hird, Abigail L. Zezeski, Thomas W. Geary, Sarah R. McCoski, El Hamidi Hay

**Affiliations:** 1 Department of Animal Science, University of Connecticut, Storrs, CT 06269, USA; 2 Institute for Systems Genomics, University of Connecticut, Storrs, CT 06269, USA; sarah.hird@uconn.edu; 3 Department of Molecular and Cellular Biology, University of Connecticut, Storrs, CT 06269, USA; 4 USDA Agricultural Research Service, Fort Keogh Livestock and Range Research Laboratory, Miles City, MT 59301, USA; abigail.zezeski@montana.edu (A.L.Z.); tgeary1909@gmail.com (T.W.G.); elhamidi.hay@usda.gov (E.H.H.); 5 Department of Animal and Range Sciences, Montana State University, Bozeman, MT 59717, USA; sarah.mccoski@montana.edu

**Keywords:** fertility, microbiome, beef cattle

## Abstract

Reproductive performance is a key determinant of overall livestock productivity. In both beef and dairy systems, reproductive failure represents a leading cause of cow culling. Reproductive traits are complex in nature and present a low heritability in general. Additionally, the collection of such phenotypes usually relies on indirect measures of fertility, such as conception success. Therefore, further investigation into genetic and non-genetic factors of reproductive traits in cattle is necessary. The hosts’ microbiome plays a crucial role in vertebrate biology, including reproduction. We, therefore, hypothesize that microbiome indicators may serve as a biomarker of fertility. This study explored the relationship between vaginal microbiome profiles and pregnancy among three beef cattle genetic groups using field data. Vaginal swabs were collected from 74 cows at Fort Keogh, MT, including 23 Angus, 23 Hereford Line 1, and 28 crossbreds, and DNA was extracted and analyzed via 16S rRNA gene amplification. Significant differences in alpha diversity (*p* < 0.05) were found among Line 1 cows compared to Angus and crossbreds in many indicators of alpha diversity. Pregnancy status did not influence alpha diversity of samples significantly, but trends toward significance were observed. PERMANOVA analysis indicated that genetic groups and pregnancy status affected microbial composition (*p* < 0.05), but their interaction was not significant. Each genetic group showed unique compositions of operational taxonomic units (OTUs), with higher proportions of *Ureaplasma* and *Mycoplasma* families in Line 1. Additionally, variations in microbial communities were observed between pregnant and non-pregnant cows, with certain uncultured bacteria more prevalent in non-pregnant cows. While field data are useful for such studies and represent a real production system, better-designed experiments are necessary to validate findings and test hypotheses. These results suggest variation in vaginal microbiomes across breeds and pregnancy status, emphasizing the need for further research to identify factors affecting these changes.

## Simple Summary

Reproductive failure is a large economic burden on cattle production; therefore, identifying factors that affect fertility is important to improve livestock profitability. In this study, vaginal microbiomes of seventy-four beef cows from three genetic groups (Angus, Hereford Line 1, and crossbreds) were examined using 16S rRNA sequencing. The Line 1 cows differed significantly from the other groups in multiple diversity measures. Moreover, pregnancy status influenced diversity within Line 1. Overall, microbial community composition was affected by genetic group and pregnancy status. Each breed had distinct OTU profiles: Line 1 had higher *Ureaplasma* and *Mycoplasma*, and some uncultured bacteria were more common in non-pregnant cows. The findings show breed- and pregnancy-associated differences in vaginal microbiomes and warrant further research to determine drivers and fertility associations.

## 1. Introduction

Fertility plays a crucial role in the profitability of beef cattle operations, with reproductive failure being a major cause of cow culling. Approximately one-third of beef cows culled from a herd are eliminated because of reproductive failure [1]. Fertility is controlled by multiple factors, such as management, environment, health, and genetics, that interact across the lifespan of the dam [2,3,4,5,6,7].

To improve fertility, genetic selection is one of the tools currently used; however, little progress has been achieved. This is mainly due to the low heritability of fertility, which ranges between 0.07 and 0.20 observed across populations and trait definitions [8,9,10,11]. Therefore, the rate of genetic change and progress is slow. Moreover, several quantitative trait loci (QTL) associated with fertility have been reported and can be found in the Animal QTL database [12]. However, overlap of significant QTL across studies is generally poor, reflecting differences in populations, environments, and marker panels, as well as small effect sizes for polygenic traits [13,14,15]. This highlights the complexity and polygenic nature of fertility in cattle.

Due to the complexity and low heritability of reproductive traits, further investigation into non-genetic factors, such as microbiomes, is warranted. Microbiomes are involved in many biological functions, including performance, health, and disease. Studies have been conducted to evaluate the effect of different microbiomes on growth, feed efficiency, and reproduction in livestock and humans [16,17]. For reproduction and fertility, the reproductive tract harbors diverse bacterial communities [18]. A common bacterium in the reproductive tract for many animals is *Lactobacillus* spp., which has been associated with maintaining the optimum pH level and controlling the overgrowth of harmful bacteria [19]. Disruptions to the reproductive tract’s microbiome, such as changes in the richness or evenness of microbial populations, have been associated with numerous reproductive diseases and infertility in humans and other species [20].

Among the many factors that affect fertility in cattle, the interaction among management, environment, and genetics remains challenging, as many confounding factors arise during sample collection. Adapted populations show selection signatures in genomic regions associated with fertility [21,22,23]. Nonetheless, drawing clear conclusions regarding the existence of genotype by environment interactions remains a challenge in beef cattle [24,25,26]. Some studies reported only marginal G × E for first pregnancy across environments, while broad literature reviews note genetic correlations < 0.80 across environmental gradients for many traits [25]. Seasonality in production systems adds another layer of complexity. However, the use of novel phenotypes allows for better modeling [27]. Additionally, biomarkers, such as microbiome features, may serve as latent phenotypes for complex traits that are difficult to measure directly [28]. Since these biomarkers play a role in biological pathways, such as immunity and hormone signaling, and are shown to be associated with fertility, they offer the potential to augment traditional fertility phenotypes by providing mechanistic insight and improving prediction accuracy in diverse environments [29].

The objective of this study was to characterize and evaluate the relationship between vaginal microbiome profiles and pregnancy status in cows and heifers after the mating season, among three beef cattle genetic groups, using field data.

## 2. Materials and Methods

### 2.1. Compliance

Samples were collected by the employees at USDA-ARS with approval from the Institutional Animal Care and Use Committee (IACUC No. 60523-1 Start date: 1 June 2023; End date: 30 May 2025).

### 2.2. Sample Collection

**Figure 1:**
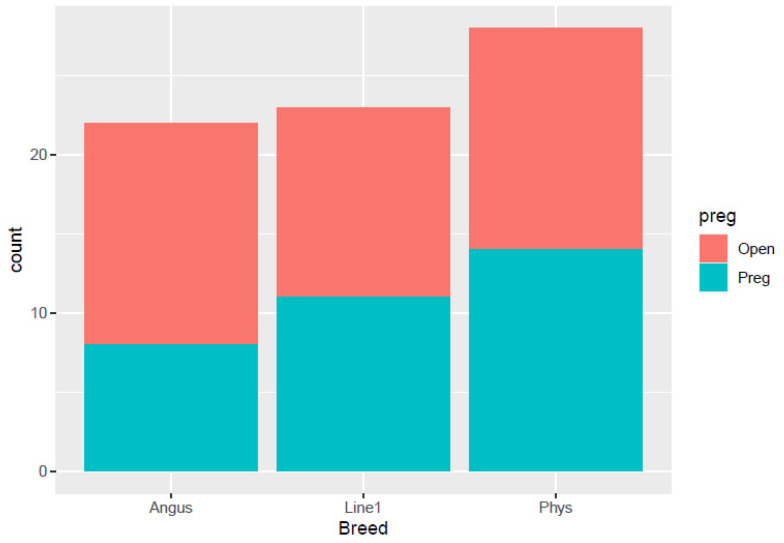
Distribution of open and pregnant cows across breeds and herds of females used for vaginal microbiome samples.

Vaginal swab samples were collected from 74 open and pregnant cows and heifers from 3 populations at the USDA Agricultural Research Service livestock and range research laboratory in Miles City, MT (46.4083° N, 105.8406° W). The populations in this study were Angus, Line 1 Hereford, and an admixed population, hereby termed the Physiology herd. Animals were under similar management practices, and all samples were collected on the same day. Three genetic groups were in different breeding pastures. No specific experimental design was made, and data were collected from animals available in the field, representing a typical beef cattle operation. Samples were collected from healthy animals without any history of reproductive or recent infectious disease. We aimed to collect an equal number of samples from pregnant and open individuals across all three genetic groups. The distribution of animals by breed and pregnancy outcome is shown in Figure 1. All animals were exposed to fertile bulls during the mating season in the months of June and July, and the pregnant individuals were in their second trimester of gestation, as determined by ultrasonography. Before sample collection, the vulva and perineum were cleaned with a 70% ethanol solution to avoid contamination. The specimens were collected using a sterile nylon swab after opening the vulva lips and inserting a disposable sterile vaginal speculum. The swabs were swirled six times in the anterior vagina before being withdrawn. Swabs were placed in sterile vials and stored at 4 °C during transport and then stored at −80 °C until sequencing.

### 2.3. DNA Extraction and Sequencing

Bacterial DNA from the vaginal swabs was extracted and purified, and Illumina library preparation was performed following the protocol used at the Microbial Analysis, Resources, and Services (MARS) Facility at the Center for Open Research Resources and Equipment at the University of Connecticut. Partial bacterial 16S rRNA genes (V4) were amplified using 30 ng of extracted DNA as a template. The V4 region was amplified using 515F and 806R with Illumina adapters and dual indices (8 base pair [30]). Samples were amplified in triplicate 15 µL reactions using Go-Taq DNA polymerase (Promega, Madison, WI, USA) with the addition of 3.3 µg of BSA (New England BioLabs, Ipswich, MA, USA). To overcome inhibition from host DNA, 0.1 pmol of primer without the indexes or adapters was added to the master mix. The PCR protocol was incubation at 95 °C for 3.5 min, then 30 cycles of 30 s at 95.0 °C, 30 s at 50.0 °C, and 90 s at 72.0 °C, followed by final extension at 72.0 °C for 10 min. PCR products were pooled for quantification and visualization using the QIAxcel DNA Fast Analysis (Qiagen, Hilden, Germany). PCR products were normalized based on the concentration of DNA from 250 to 400 bp then pooled using the epMotion 3075 liquid handling robot (Eppendorf, Hamburg, Germany). The pooled PCR products were cleaned using Omega Bio-Tek Mag-Bind Beads (Omega Bio-Tek, Norcross, GA, USA) according to the manufacturer’s protocol using 0.8× beads in the PCR product. The cleaned pool was sequenced on the MiSeq using v2 2 × 250 base pair kit (Illumina, Inc., San Diego, CA, USA) according to the manufacturer’s instructions. Low-quality reads were filtered, and the data were trimmed to a consistent length for the forward and reverse reads. The remainder were used to classify each read into an operational taxonomic unit (OTU), based on the similarity between our sample and the database, using the open-source software Mothur v1.48.2 [31]. Data were uploaded to R (version 4.5.2) using the phyloseq package version 1.54.2 [32].

### 2.4. Statistical Analysis

All analyses were conducted using R version 4.5.2 [33] and the packages phyloseq version 1.54.2 [32] and microbiome version 1.23.1 [34]. For quality control, OTUs with total counts across all samples lower than 100 were removed to increase the reliability of the microbiome composition [35]. Additionally, samples with a Shannon alpha diversity lower than 0.40 were removed, since they were more than 3.5 standard deviations from the mean and represented outliers. Finally, samples were rarefied to 2000 reads to standardize the uneven sequencing depth. This sequence depth maximizes the number of individuals in the study.

Twenty-two different metrics of alpha diversity were calculated (Table 1). Differences between effects of genetic group, pregnancy status, birth year, and number of calves produced previously were tested under a linear assumption. Moreover, a Wilcoxon signed-rank test was used across pairs of effects. All *p*-values were adjusted for multiple comparisons using the Benjamini–Hochberg (HB) false discovery rate correction [36]. The following linear model was fit:

$$y_{ij}=Status_{i}+e_{ij},$$

where $y_{ij}$ is the microbiome metric (i.e., Shannon diversity index) of cow *j* with pregnancy status *i*. Later, status was substituted by genetic group, birth year, and number of calves using a similar model. Additionally, pregnancy status and genetic group were tested together. Finally, a model that also included the interaction between the two effects was fitted.

The beta diversity was calculated by grouping cows into pregnant and open, and by each genetic group. Further, a model including both breed and pregnancy status was fitted. Using a principal coordinate analysis of the Bray–Curtis dissimilarity distances, the sample types were mapped into groups to assess the distance between the centers of the clusters. The associations were evaluated under a one-way PERMANOVA [37] analysis using the adonis2 command of the R package vegan version 2.4-4 [38].

The abundances of the OTUs in the different groups were compared by a visual inspection of a composition plot of the relative abundances. Taxonomic classification was performed against the Silva database release 119 [39], and the taxonomic rank of family was used. Finally, a differential abundance analysis was performed using a pairwise comparison of the variance of abundance. The differential bacterial abundance was identified by the Wald test in the DEseq2 R package version 1.40.2 [40] and the *p*-values were adjusted using the DH FDR correction.

## 3. Results and Discussion

### 3.1. Distribution

**Figure 2:**
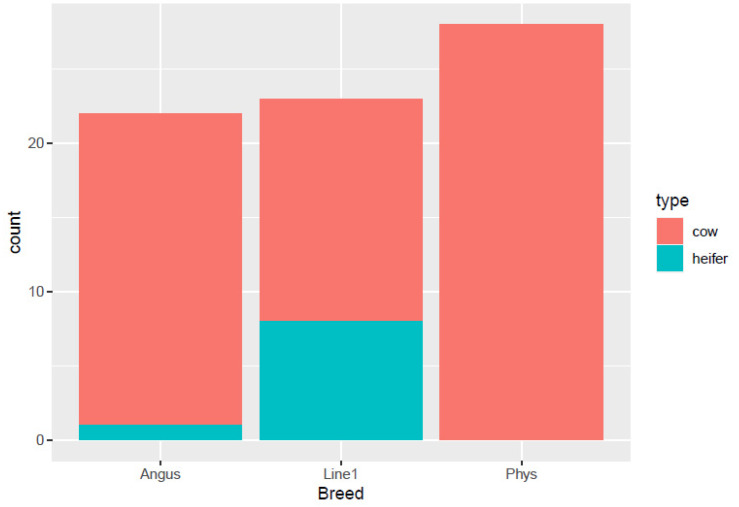
Distribution of cows and heifers across Angus, Hereford Line 1 (Line1), and Physiology (Phys) populations sampled for vaginal microbiome.

The distribution of pregnancy status (open or pregnant) among different cow breeds: Angus, Line 1, and Physiology herd, is shown in Figure 1. Line 1 had equal pregnant cows (*n* = 9) compared to open ones (*n* = 9). The Physiology herd had approximately equal numbers of open (*n* = 12) and pregnant cows (*n* = 10), while Angus had more open cows (*n* = 12) than pregnant cows (*n* = 8). For the distribution of cows and heifers in each breed (Figure 2), the Physiology herd consisted of all cows, and the Angus had only one heifer. On the other hand, Line 1 had more heifers compared to the other breeds.

### 3.2. Number of Reads and Rarefication

The number of reads varied from 578 to 144,255. No association between the number of reads and any genetic group or pregnancy status was found. The rarefication at 2000 reads maximizes the number of viable samples after quality control to 60 (Supplementary Figure S1). While there are arguments in favor of and against rarefication [41,42], it is undeniable that the variation in the number of reads observed in our samples would require some sort of preprocessing. However, such preprocessing resulted in a smaller sample size and power. Since the reproductive tract is a low-biomass sampling site, such problems are expected to occur [43]. A solution for future studies is to use specific DNA extraction kits for low-biomass sites [44].

### 3.3. Alpha Diversity

For the alpha diversity results, the statistical significance of all indicators is included in Table 1. Raw values for alpha diversity indices and the effect size for all analyses are included in Supplementary Table S1. The results were similar using the linear model and the two-sample Wilcoxon test; therefore, only the two-sample Wilcoxon test results are presented for pairwise comparisons. The *p*-values for pregnancy status for the combined population were all non-significant. When pregnant vs. open cows’ microbiomes were compared within breeds, six features were statistically different within the Line 1 population before HB FDR correction. However, adjusted *p*-values were not significant for this test. No differences were found within the Physiology and Angus animals. A study by Webb et al. [45] found that beef heifers that successfully conceived after artificial insemination had a distinct vaginal microbiome compared to those that remained open. However, similar to the present study, they did not find a significant difference between pregnant and open cows.

The cow and heifer comparison was only performed within the Line 1 population, since the other two did not have enough heifers. This comparison did not yield any significant differences in the microbiome alpha diversity.

In contrast to pregnancy status, genetic groups had a significant effect on the microbiome alpha diversity. The differences between breeds were significant for most alpha diversity metrics using the linear model. The main difference was between Line 1 animals and the other two populations, which is illustrated in Table 1. No differences were observed between Angus and Physiology herds. This could potentially be due to the high inbreeding and closed population structure of Line 1. However, since the animals were in different breeding pastures, the breed and pasture effects were confounded. Since no differences were observed between the Physiology and Angus herds, we hypothesize a potential effect associated with Line 1 genetics. A study by Teng et al. [46] reported no significant differences in vaginal microbiome between the two Chinese cattle breeds, Yanbian and Yanhuang cattle. These two Chinese cattle breeds are genetically similar (Yanhuang is composed of 75% Yanbian), as is the case with Angus and the Physiology herds. Another study examining vaginal microbiome differences between Holstein and Fleckvieh cattle found a significantly higher Shannon index in Fleckvieh cattle compared to Holstein cattle [47]. These differences in microbiome diversity across breeds suggest an underlying genetic mechanism and may be due to the degree of genetic similarity or dissimilarity between the breeds. Genetic aspects of within-breed variation would require a larger sample size and genomic information or pedigree on the animals [48]. Moreover, to model across-breed effects, an experimental design that randomizes animals across pastures would be ideal. However, since exploratory field data were used in this study, the breed and pasture contrast could not be tested.

A linear model that included both genetic group and pregnancy effect yielded similar results as the effects fitted separately (Supplementary Table S2). In an additional model, all interactions between genetic groups and pregnancy status were not significant (Supplementary Table S3). Due to the limited sample size and unbalanced data structure, overparametrized models resulted in reduced fit when *p*-values were adjusted for the number of parameters. However, the model that fitted together genetic groups and pregnancy status resulted in lower *p*-values for the pregnancy status, approaching the arbitrary 0.05 threshold and demonstrating a trend toward significance.

### 3.4. Beta Diversity

**Figure 3:**
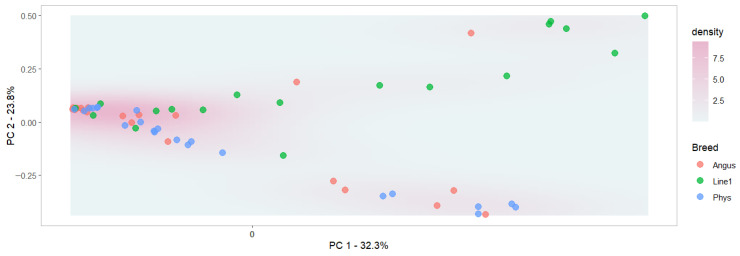
Principal coordinate analysis for the beta diversity plot of the microbiome composition. Each dot represents a vaginal microbiome sample categorized into breed: Angus, Hereford Line 1 (Line1), and Physiology (Phys). The percentage of variance explained by the first and second PCs is included on the *x* and *y* axes, respectively.

**Figure 4:**
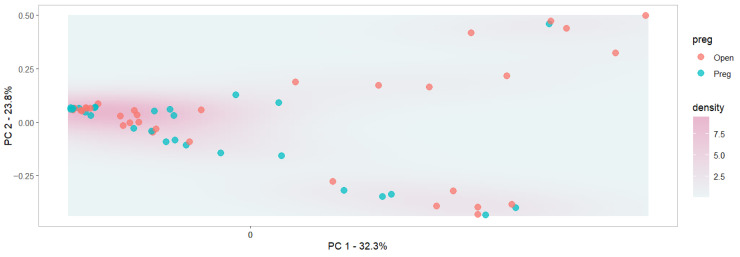
Principal coordinate analysis for the beta diversity plot of the microbiome composition. Each dot represents a vaginal microbiome sample categorized into pregnant status (preg). The percentage of variance explained by the first and second PCs is included on the *x* and *y* axes, respectively.

Beta diversity metrics are used to assess the dissimilarity between samples and groups. Based on beta diversity testing, ordination revealed clustering that suggests microbiomes segregate based on breeds (Figure 3; *p* < 0.01), but not between open and pregnant cows (Figure 4; *p* = 0.14). This indicates that there are significant differences in the microbiome of different subpopulations, but not based on pregnancy status, when breeds are not taken into consideration. Pregnancy status within the Line 1 population suggested microbiome segregation between pregnant and open cows (*p* < 0.05). No significance was observed between pregnant and open cows within the Angus and Physiology populations. The model that included the genetic group and pregnancy status was significant (*p* < 0.001), as was the model that included the interaction between the two terms. The R^2^ values of the models with genetic groups only, the inclusion of the pregnancy status, and the interaction were, respectively, 0.11, 0.15, and 0.19. The adjusted R^2^ values followed the same trend: 0.07, 0.09, and 0.12, respectively, indicating a better fit, suggesting that beta diversity differs across those groups and with their interaction. However, those values are small and indicate that many other factors contribute to microbiome variation. Ault et al. [49] reported differences in beta diversity in the vaginal microbiome between pregnant and non-pregnant cows prior to artificial insemination. It is important to mention that the differences observed in the current study were after pregnancy was established and could reflect hormonal changes during the pregnancy of animals [50]. Similar to our findings, Webb et al. [45] reported a significant difference in beta diversity between pregnant and open heifers.

The results of the beta diversity suggest that the relationship between microbiome and fertility may be breed-specific. Another possibility is that, because Line 1 has a more uniform population and exhibits high homozygosity, a smaller sample size was sufficient to detect microbiome differences.

The PERMANOVA results agreed with those of the alpha diversity, and identified significant differences between breeds, but not between pregnant and open cows. This supports that the breed is an indicator of microbial composition. PERMANOVA for type within the Line 1 population was tested, and no significant difference between heifers and cows was observed.

### 3.5. Microbial Composition

**Figure 5:**
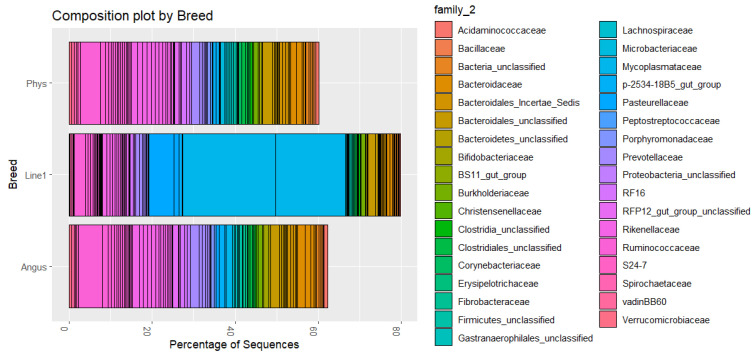
Relative abundance (percentage of sequences) of OTU family composition of vaginal microbiome samples merged within breed: Angus, Hereford Line 1 (Line1), and Physiology (Phys). The top 100 OTUs were selected for this graph.

**Figure 6:**
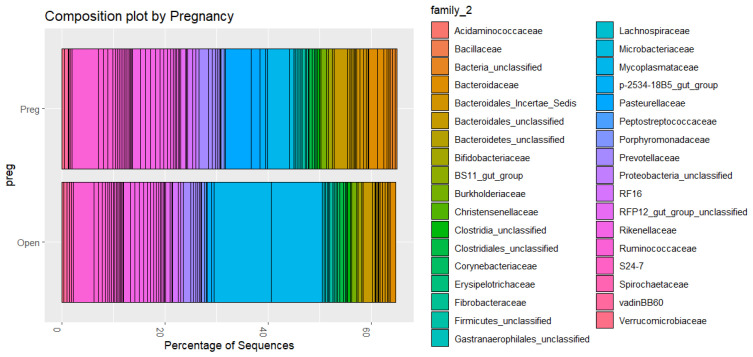
Relative abundance (percentage of sequences) of OTU family composition of vaginal microbiome samples merged within pregnancy status. The top 100 OTUs were selected for this graph.

The composition plots included the 100 most common OTU families. The relative abundance of the top families accounted for 60% to 80% of the microbiome. Visual inspection of the composition plots in Figure 5 showed differences between Line 1 and the other two genetic groups. The main differences were the higher composition of Mycoplasmataceae (with two OTUs represented), Ruminococcaceae, an unclassified Bacteroidales, and Pasteurellaceae. Similarly, between open and pregnant cows, visual inspection of Figure 6 showed a higher composition of the same two Mycoplasmataceae OTUs and Ruminococcaceae in open animals, and a higher composition of Pasteurellaceae in pregnant animals. Studies have shown various dominant bacterial phyla and genera in vaginal microbiome. Laguardia-Nascimento et al. [51] characterized the vaginal microbiome of healthy Nellore, and identified Firmicutes, Bacteroidetes, and Proteobacteria as the main phyla, and the dominant genera included *Aeribacillus*, *Clostridium*, and *Prevotella*. Firmicutes and Bacteroidetes were the most abundant phyla in postpartum Angus cows prior to artificial insemination [49]. However, the most abundant genera were not identified from Family Ruminococcaceae and other non-identified genera in the Order Clostridiales. In the present study, the most abundant phyla in Line 1 were *Tenericutes*, followed by *Bacteroidetes* and *Firmicutes*. The top OTUs across the three genetic groups belonged to similar Phyla as those identified as the ‘core’ vaginal microbiome in dairy cattle—*Tenericutes*, for instance, were among the most common OTUs in healthy heifers [52]. For the other two populations, Bacteroides and Firmicutes were the most abundant phyla, which may indicate a unique characteristic of Line 1 animals, or a potential dysbiosis, since *Mycoplasma* may be associated with bacterial vaginosis in humans [53]. However, *Mycoplasma* can also be endemic in bovines, causing mastitis in [54] and respiratory diseases [55]. There are approximately 30 *Mycoplasma* species identified in cattle, and only a few of them are known to be pathogenic [56]. Similarly, some species in the *Ureaplasma* genus can be commensal in cattle, while others, such as *U. diversum*, can be associated with reproductive disorders [57]. However, 16S sequencing does not have the resolution to identify species [58]. Therefore, higher-resolution methods are necessary to identify the exact species and determine their functions. Additionally, the animals in the present study were in the second trimester of gestation; therefore, comparison with open cows or postpartum cows must be made cautiously, since even a period of 20 days can promote differences in the vaginal microbiome [49].

**Figure 7:**
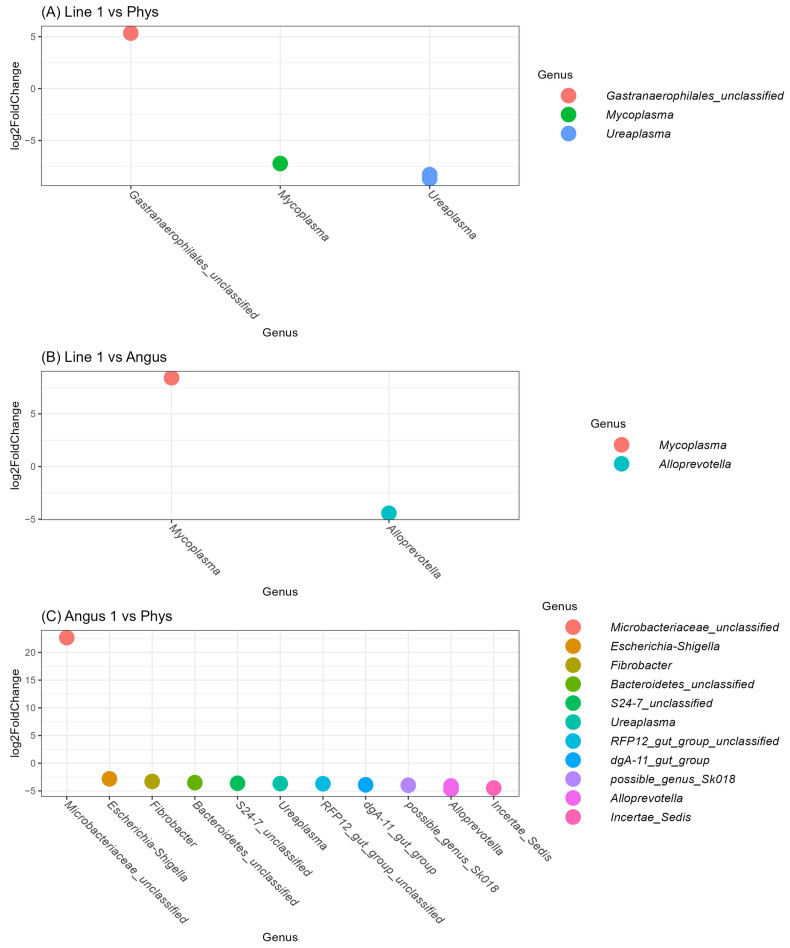
Pairwise comparison of variation in abundance using DESeq2. The figures represent the significant differences in the log2 fold change in OTUs between breeds: (**A**) Angus and Physiology, (**B**) Hereford Line 1 and Angus, and (**C**) Hereford Line 1 and the Physiology herd. Values were considered statistically significant if *p*-values adjusted by the Benjamini–Hochberg false discovery rate procedure were below 0.05.

Based on the differential expression analysis, we identified that the two Mycoplasmaceace were significantly different between Line 1 and Physiology animals, represented by the genera *Mycoplasma* and *Ureaplasma* in Figure 7A. Additionally, two new OTUs were significantly different and were not identified in the top 100 OTUs: an unclassified Gastranaerophilales and an additional *Mycoplasma*. Between Line 1 and Angus animals, the *Mycoplasma* included in the top 100 OTU was also significant, in addition to an *Alloprovotella* OTU, as shown in Figure 7B. Figure 7C shows that 12 OTUs were significantly different between Angus and Physiology animals, including the *Ureaplasma* previously described and OTUs not included in the top 100 previously described. Two *Ureaplasma* and a *Corynebacterium* were identified.

**Figure 8:**
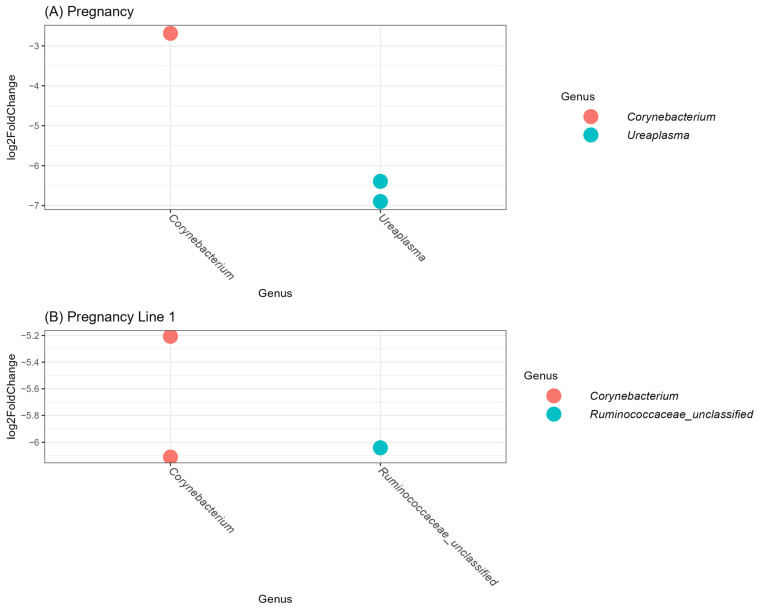
Pairwise comparison of variation in abundance using DESeq2. The figures represent the significant differences in the log2 fold change in OTUs between pregnant and open animals: (**A**) across all breeds and (**B**) within the Hereford Line 1 population. Values were considered statistically significant if *p*-values adjusted by the Benjamini–Hochberg false discovery rate procedure were below 0.05.

Three OTUs were significantly different between pregnant and open animals across all animals (Figure 8A). Fewer differences between pregnant and open animals within breed were observed. For the Angus population, only a *Chlamydophila* was identified, and no differences were observed in the Physiology herd. Finally, the Line 1 population differed on two Corynebacteriaceae and an unclassified Ruminococcaceae OTU (Figure 8B).

The findings in the present study suggest that the vaginal microbiome varies significantly across breeds or genetic groups. Sanglard et al. [48] estimated heritabilities for 100 OTUs in vaginal samples from gilts, and found moderate to high heritability in many of them. Moreover, QTLs associated with such OTUs were identified, indicating a genetic component explaining the host microbiome relationship in pigs. Additionally, Sanglard et al. [59] identified differences between high- and low-fertility sows, indicating that individual fertility differences among animals may be linked to genetics and microbiome interaction. In cattle, host–microbiome relationships are well studied, especially regarding the digestive tract microbiome [60]. Additionally, heritable microbiota can be associated with dairy performance [29] and feed efficiency [61], suggesting that genetic tools, such as genomic selection, could be applied to improve such traits through microbiome manipulations [62]. While the current study did not find associations with pregnancy, the vaginal microbiome in ruminants is highly diverse [63] and was previously associated with fertility [51]. Therefore, increasing the sample size and identifying the ideal sampling period are necessary to establish a relationship between the vaginal microbiome and fertility and to implement a genomic selection program for fertility in cattle to improve fertility through manipulation of the vaginal microbiome. While stronger associations were observed in uterine swabs, those are more invasive and may be challenging to collect on a large scale.

### 3.6. Limitations and Future Steps

The present study investigated associations between genetic groups, pregnancy status, and the vaginal microbiome in beef cattle using field data. Confounding factors are present in our experimental design, and we should exercise caution before drawing major conclusions. The pregnant animals were in their second trimester, so the observed differences could indicate that cows with established pregnancies differ from open cows. To make a stronger claim regarding fertility, animals should be sampled right before artificial insemination or in the following weeks, while pregnancy is being established. Other effects, such as animal age, status (heifer/cow), and number of calves, among others, could not be properly tested due to unbalanced distribution in our data. Another limiting factor is that the estrous stage of open cows may affect the microbiome. Such an effect was not tested in our study and could have contributed to a large variation in open animals, decreasing the detection power of our associations for fertility.

The three genetics groups were managed the same way but in different pastures. Therefore, the genetic group difference is confounded with pasture. An improved experimental design is needed to draw final conclusions regarding the effects of genetics on the vaginal microbiome. However, since Line 1 Hereford had a unique microbiome, we hypothesize that such differences can be attributed to its genetics. Such a hypothesis must be tested with a 2 × 2 factorial design, for instance. Additionally, within-breed genetic contribution to the microbiome could be investigated with a larger sample size. The Line 1 Hereford population [64,65], included in the present study, poses an attractive solution to genome–microbiome integration studies. The results suggest that a unique microbiome composition may exist in this breed. Additionally, due to its small effective population size [64,65], fewer independent chromosome segments need to be estimated [66], which maximizes the discovery power of genomic studies [67].

## 4. Conclusions

Diversity and composition of the vaginal microbiome varied significantly across different genetic groups. Alpha and beta diversity illustrated mostly the differences between Line 1 animals and the Physiology and Angus populations. The microbiome composition differed significantly across genetic groups, with more differences observed between Angus and Physiology cows. Fewer differences were observed regarding the pregnancy status of animals, and most of these microbiome variations were observed within Line 1 animals. Such findings suggest that there is a strong breed component of the vaginal microbiome, and the association between fertility and microbiome composition may vary within genetic groups. Finally, this study illustrates that while field data may provide important results, better-designed experiments are necessary for further investigation of the microbiome of the reproductive tract and its relationship with fertility.

## Figures and Tables

**Table 1:** Significance for 22 alpha diversity indices across different groups. Pregnancy Line 1 indicates differences across open and pregnant Line 1 animals with a pairwise Wilcoxon model. Linear genetic group was fitted as a linear effect including the three populations. The last three columns contain the pairwise Wilcoxon model comparing Angus and Physiology, Line 1 and Physiology, and Line 1 and Angus animals, respectively. Values with a * indicate significant differences at *p* < 0.05. *p*-values were adjusted using the Benjamini–Hochberg false discovery rate correction.

Index	Pregnancy Line 1	Linear Genetic Group	Angus vs. Phys	Line 1 vs. Phys	Line 1 vs. Angus
observed	0.100	0.004 *	0.990	0.008 *	0.017 *
chao1	0.100	0.018 *	0.720	0.057	0.029 *
diversity_inverse_simpson	0.100	0.013 *	0.510	0.003 *	0.026 *
diversity_gini_simpson	0.100	0.002 *	0.510	0.003 *	0.026 *
diversity_shannon	0.100	0.002 *	0.900	0.002 *	0.017 *
diversity_fisher	0.100	0.004 *	0.990	0.008 *	0.017 *
diversity_coverage	0.102	0.003 *	0.656	0.001 *	0.017 *
evenness_camargo	0.100	0.002 *	0.510	0.000 *	0.017 *
evenness_pielou	0.100	0.002 *	0.510	0.002 *	0.018 *
evenness_simpson	0.331	0.018 *	0.510	0.003 *	0.042 *
evenness_evar	0.331	0.510	0.990	0.712	0.729
evenness_bulla	0.100	0.002 *	0.510	0.000 *	0.017 *
dominance_dbp	0.100	0.002 *	0.510	0.004 *	0.033 *
dominance_dmn	0.100	0.002 *	0.510	0.003 *	0.026 *
dominance_absolute	0.100	0.002 *	0.510	0.004 *	0.033 *
dominance_relative	0.100	0.002 *	0.510	0.004 *	0.033 *
dominance_simpson	0.100	0.002 *	0.510	0.003 *	0.026 *
dominance_core_abundance	0.100	0.002 *	1.000	0.004 *	0.021 *
dominance_gini	0.100	0.003 *	0.989	0.003 *	0.017 *
rarity_log_modulo_skewness	0.183	0.400	0.510	0.804	0.345
rarity_low_abundance	0.100	0.013 *	0.989	0.032 *	0.026 *
rarity_rare_abundance	0.318	0.808	0.510	0.199	0.363

## Data Availability

Dataset available upon request from the authors.

## Supplementary Materials

The following supporting information can be downloaded at: https://www.mdpi.com/article/10.3390/ani16060874/s1, Figure S1: Rarefaction curve showing the number of OTUs in the *y* axis and the number of reads in the *x* axis from 74 vaginal microbiome samples collected in pregnant and open beef cows and heifers from Hereford Line1, Angus, and Physiology populations; Table S1: Mean, standard deviation (sd), and effect estimates for 22 alpha diversity indices across different groups; Table S2: Significance for 22 alpha diversity indices in a linear model including all three genetic groups and pregnancy status; Table S3: Significance for 22 alpha diversity indices in a linear model including all three genetic groups, pregnancy status, and their interaction.

## Abbreviations

The following abbreviations are used in this manuscript:

OTU	Operational Taxonomic Unit
QTL	Quantitative Trait Loci
BH	Benjamini–Hochberg
FDR	False Discovery Rate
Preg	Pregnancy
SD	Standard Deviation
