# Supplementary material for: Vaginal Microbiome Is Associated with Breed and Pregnancy Status in Beef Cattle
Source: Animals (Basel). 2026 Mar 11;16(6):874. doi: 10.3390/ani16060874 (PMC13023300; doi:10.3390/ani16060874)

Supplementary Figure S1. Rarefaction curve showing the number of OTUs in the *y* axis and the number of reads in the *x* axis from 74 vaginal microbiome samples collected in pregnant and open beef cows and heifers from Hereford Line1, Angus, and Physiology populations

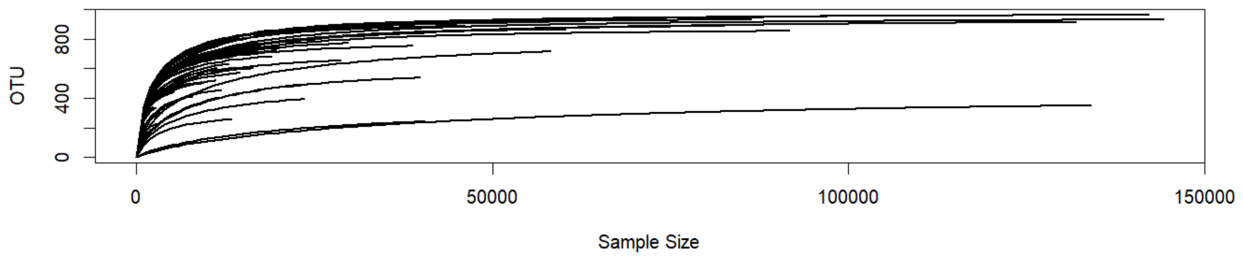

Supplement: Supplementary file 1 [file animals-16-00874-s001.zip › Supplementary Figure S1.pdf]
