# Supplementary material for: Vaginal Microbiome Is Associated with Breed and Pregnancy Status in Beef Cattle
Source: Animals (Basel). 2026 Mar 11;16(6):874. doi: 10.3390/ani16060874 (PMC13023300; doi:10.3390/ani16060874)
Supplement: Supplementary file 1 [file animals-16-00874-s001.zip › Supplementary Table S1.pdf]

Supplementary Table S1. Mean, standard deviation (sd), and effect estimates for 22 alpha diversity indices across different groups. The estimates were calculated under different linear models. The effects in the table are the deviation from the intercept for the estimable contrasts. The effects tested included the effects of pregnancy across all populations (Pregnancy); or the pregnancy only for Hereford Line1 (Pregnancy Line1). The genetic group effects (Breed) were calculated under a model that included all three genetic groups. An additional effect that included genetics groups and pregnancy (Breed + Pregnancy was also tested). Finally, a model that included the genetic groups, pregnancy, and their interaction was tested (Breed + Pregnancy with interaction). The interactions are represented with the ':' symbol.

			Pregnancy	*Pregnancy Line1*	*Breed*		*Breed + Pregnancy*			*Breed + Pregnancy with Interaction*				
	mean	sd	preg	preg line1	Breed Line1	Breed Phys	BreedLine1	BreedPhys	Preg	BreedLine1	BreedPhys	Preg	Line1:Preg	Phys:Preg
observed	347.19	111.27	47.136	126.88	-108.78	14.771	-117.270	9.491	57.706	-154.20	35.709	51.076	75.813	-57.626
chao1	581.35	196.26	57.566	187.36	-190.24	-34.396	-201.499	-41.402	76.564	-253.00	16.620	87.579	99.781	-132.818
diversity_inverse_simpson	60.69	42.37	9.503	29.545	-34.537	14.057	-36.396	12.901	12.639	-43.525	23.288	16.390	13.155	-24.144
diversity_gini_simpson	0.88	0.22	0.083	0.254	-0.231	0.035	-0.246	0.025	0.106	-0.339	0.053	0.054	0.201	-0.053
diversity_shannon	4.47	1.41	0.588	1.609	-1.447	0.304	-1.553	0.238	0.726	-2.059	0.499	0.543	1.065	-0.551
diversity_fisher	151.01	62.89	23.265	62.011	-61.528	8.754	-65.820	6.084	29.186	-81.648	22.082	30.842	31.169	-36.337
diversity_coverage	31.11	19.14	6.636	15.667	-17.265	6.513	-18.476	5.759	8.240	-21.242	11.091	10.924	4.742	-12.549
evenness_camargo	0.32	0.10	0.038	0.109	-0.095	0.034	-0.102	0.030	0.047	-0.140	0.045	0.029	0.080	-0.031
evenness_pielou	0.76	0.21	0.088	0.246	-0.221	0.047	-0.237	0.037	0.109	-0.318	0.072	0.073	0.173	-0.072
evenness_simpson	0.15	0.09	0.022	0.066	-0.073	0.036	-0.077	0.033	0.029	-0.094	0.055	0.035	0.032	-0.049
evenness_evar	0.56	0.07	0.028	0.072	-0.016	0.004	-0.021	0.001	0.030	-0.049	0.005	0.010	0.061	-0.005
evenness_bulla	0.48	0.14	0.064	0.170	-0.135	0.036	-0.146	0.029	0.077	-0.202	0.050	0.050	0.120	-0.043
dominance_dbp	0.21	0.26	-0.094	-0.270	0.263	-0.063	0.281	-0.052	-0.119	0.368	-0.096	-0.086	-0.184	0.091
dominance_dmn	0.26	0.26	-0.103	-0.291	0.267	-0.062	0.286	-0.050	-0.128	0.377	-0.101	-0.098	-0.193	0.108
dominance_absolute	316.60	388.40	-141.094	-405.11	394.85	-94.477	421.093	-78.152	-178.409	551.909	-143.491	-128.924	-276.18	136.824
dominance_relative	0.21	0.26	-0.094	-0.270	0.263	-0.063	0.281	-0.052	-0.119	0.368	-0.096	-0.086	-0.184	0.091
dominance_simpson	0.12	0.22	-0.083	-0.254	0.231	-0.035	0.246	-0.025	-0.106	0.339	-0.053	-0.054	-0.201	0.053
dominance_core_abundance	0.40	0.15	0.053	0.155	-0.172	0.019	-0.182	0.013	0.070	-0.239	0.022	0.030	0.125	-0.013
dominance_gini	0.95	0.02	-0.009	-0.022	0.022	-0.006	0.024	-0.005	-0.011	0.029	-0.010	-0.012	-0.010	0.013
rarity_log_modulo_skewness	2.06	0.00	0.000	0.000	0.000	0.000	0.000	0.000	0.000	0.000	0.000	0.000	0.000	0.000
rarity_low_abundance	0.20	0.06	0.025	0.067	-0.057	0.006	-0.061	0.003	0.030	-0.081	0.017	0.026	0.042	-0.030
rarity_rare_abundance	0.36	0.15	0.056	0.145	-0.007	0.021	-0.016	0.016	0.057	-0.067	0.039	0.035	0.110	-0.046
