# Supplementary material for: Vaginal Microbiome Is Associated with Breed and Pregnancy Status in Beef Cattle
Source: Animals (Basel). 2026 Mar 11;16(6):874. doi: 10.3390/ani16060874 (PMC13023300; doi:10.3390/ani16060874)
Supplement: Supplementary file 1 [file animals-16-00874-s001.zip › Supplementary Table S2.pdf]

Supplementary Table S2. Significance for 22 alpha diversity indices in a linear model including all three genetic groups and pregnancy status. Values with a * indicate significant differences at $p<0.05$. P-values were adjusted using the Benjamini-Hochberg false discovery rate correction.

	Line1	*Phys*	*Preg*
observed	0.001194*	0.889909	0.085915
chao1	0.002406*	0.889909	0.151904
diversity_inverse_simpson	0.007792*	0.889909	0.232965
diversity_gini_simpson	0.000662*	0.889909	0.085915
diversity_shannon	0.000662*	0.889909	0.085915
diversity_fisher	0.001215*	0.889909	0.085915
diversity_coverage	0.00222*	0.889909	0.101112
evenness_camargo	0.000662*	0.889909	0.085915
evenness_pielou	0.000662*	0.889909	0.085915
evenness_simpson	0.011394*	0.889909	0.232965
evenness_evar	0.385936	0.968597	0.141971
evenness_bulla	0.000662*	0.889909	0.085915
dominance_dbp	0.000662*	0.889909	0.085915
dominance_dmn	0.000662*	0.889909	0.085915
dominance_absolute	0.000662*	0.889909	0.085915
dominance_relative	0.000662*	0.889909	0.085915
dominance_simpson	0.000662*	0.889909	0.085915
dominance_core_abundance	0.000662*	0.889909	0.085915
dominance_gini	0.001194*	0.889909	0.085915
rarity_log_modulo_skewness	0.10177*	0.968597	0.114495
rarity_low_abundance	0.002901	0.953206	0.085915
rarity_rare_abundance	0.765262	0.889909	0.205476
