# Supplementary material for: Vaginal Microbiome Is Associated with Breed and Pregnancy Status in Beef Cattle
Source: Animals (Basel). 2026 Mar 11;16(6):874. doi: 10.3390/ani16060874 (PMC13023300; doi:10.3390/ani16060874)
Supplement: Supplementary file 1 [file animals-16-00874-s001.zip › Supplementary Table S3.pdf]

Supplementary Table S3. Significance for 22 alpha diversity indices in a linear model including all three genetic groups, pregnancy status, and their interaction. Values with a * indicate significant differences at p<0.05. P-values were adjusted using the Benjamini-Hochberg false discovery rate correction.

	Line1	*Phys*	*Preg*	*Line1:Preg*	*Phys:Preg*
observed	0.001*	0.596	0.599	0.378	0.779
chao1	0.003*	0.903	0.599	0.504	0.779
diversity_inverse_simpson	0.016*	0.584	0.599	0.643	0.779
diversity_gini_simpson	0.001*	0.650	0.686	0.378	0.779
diversity_shannon	0.001*	0.584	0.599	0.378	0.779
diversity_fisher	0.002*	0.584	0.599	0.504	0.779
diversity_coverage	0.006*	0.584	0.599	0.671	0.779
evenness_camargo	0.001*	0.584	0.599	0.378	0.779
evenness_pielou	0.001*	0.584	0.599	0.378	0.779
evenness_simpson	0.020*	0.584	0.599	0.643	0.779
evenness_evar	0.119*	0.903	0.763	0.378	0.917
evenness_bulla	0.001*	0.584	0.599	0.378	0.779
dominance_dbp	0.001*	0.584	0.599	0.378	0.779
dominance_dmn	0.001*	0.584	0.599	0.378	0.779
dominance_absolute	0.001*	0.584	0.599	0.378	0.779
dominance_relative	0.001*	0.584	0.599	0.378	0.779
dominance_simpson	0.001*	0.650	0.686	0.378	0.779
dominance_core_abundance	0.001*	0.770	0.687	0.378	0.915
dominance_gini	0.002*	0.584	0.599	0.504	0.779
rarity_log_modulo_skewness	0.039*	0.903	0.687	0.378	0.915
rarity_low_abundance	0.003*	0.650	0.599	0.406	0.779
rarity_rare_abundance	0.331	0.692	0.687	0.418	0.779
